# Long-Term Cardiovascular Outcomes in Childhood Cancer Survivors: A Systematic Review

**DOI:** 10.7759/cureus.85670

**Published:** 2025-06-09

**Authors:** Abdulla Fahmi, Fathima Safa, Sheza Mariya, Akash Deep, Amal C S, Adwaith S Mohan

**Affiliations:** 1 General Medicine, Malabar Medical College, Kozhikode, IND; 2 Cardiology, Malabar Medical College, Kozhikode, IND

**Keywords:** ambulatory blood pressure monitoring, cardiovascular disease, cardiovascular risk factors (cvrf), graft-versus-host disease, hematopoietic stem cell transplantation (hsct)

## Abstract

In recent years, childhood cancer appears to have become more common. Cardiovascular (CV) diseases have been cited as the leading cause of noncancer mortality among childhood cancer survivors. This systematic review evaluated the long-term CV outcomes of childhood cancer survivors. Eleven studies, published between 2015 and 2024 that satisfied the criteria for a thorough assessment, are included in the study. The research found consistent associations between specific cancer treatments, such as anthracyclines, radiation therapy, and total body irradiation, and increased risks of cardiomyopathy, coronary artery disease, and metabolic syndrome. Hematological malignancies, such as childhood acute lymphoblastic leukemia, and survivors of solid tumors like prostate and lung cancers were more specifically associated with CV complications. Although factors like obesity, hypertension, and insulin resistance were commonly reported, discrepancies based on race and gender were diverse across studies. Case in point, cardiometabolic challenges were more prevalent among non-Hispanic Black and Hispanic survivors, but these patterns were not uniform in all cohorts. Comparably, vulnerabilities such as peripartum cardiomyopathy were more evident in female survivors, particularly at younger ages and with higher anthracycline doses. These findings underscore both consistent and variable patterns of CV risks among childhood cancer survivors. The threats are influenced by the kind of cancer, the type of therapy, and demographic factors such as age, gender, and ethnicity. Thus, there is a need to put in place systematic, lifelong programs for cardiometabolic screening and risk reduction that are tailored to each person’s risk profile. These programs should include echocardiography, regular serum biomarker testing, lifestyle modifications (exercise, nutrition), and psychosocial support.

## Introduction and background

The last few decades have seen a substantial increase in childhood cancer. This increase can be attributed to lifestyle changes, diets, and better reporting [1]. The World Health Organization reports that, globally, there are about 400,000 new cases of cancer among children aged 19 and below annually [2]. Notably, however, there has been a significant advancement in cancer treatments within the same timeline. Consequently, the survival rate among children has seen significant improvement, with a five-year survival rate being as high as 90% in some first-world countries [3].

While the increase in survival rates is desirable, it also predisposes children to a high number of complications. Childhood cancer survivors face a range of late effects beyond recurrence, which include cardiovascular disease (CVD), endocrine disorders, secondary malignancies, neurocognitive deficits, and psychosocial complications [4]. However, among these, CVD has emerged as the leading cause of noncancer-related mortality [5]. For example, several echocardiography investigations have shown that some childhood cancer survivors experience subclinical cardiotoxicity, which may evolve to heart failure over time [6]. As a result, CVD has been cited as the leading cause of noncancer mortality among childhood cancer survivors [7].

The drugs and procedures involved in cancer treatment can significantly damage healthy heart and blood cells that have limited ability to regenerate [8]. According to Armenian et al., treatments such as anthracycline-based chemotherapy and chest-directed radiation therapy are well-established contributors to cardiotoxicity, damaging cardiac myocytes and vascular endothelium, both of which have limited capacity to regenerate [6]. That inability to regenerate is the main reason why childhood cancer survivors are exposed to certain conditions that may only become evident after several years, sometimes decades.

Other than the treatment, additional factors such as cumulative drug doses and genetic predisposition influence the level of exposure to CVDs. According to Lipshultz et al., childhood cancer survivors are at least seven times more likely to suffer cardiac-related premature death than the general population [9]. These CV effects may not appear immediately. In fact, several large cohort studies have shown that clinically significant cardiac events, depending on treatment type and intensity, often emerge between 20 and 30 years posttreatment [10].

Some of the cardiac diseases that have been shown to exhibit a high correlation with survival of childhood cancer include heart failure, coronary artery disease, valvular heart disease, pericardial disease, and arrhythmia. According to Armstrong et al., left ventricular systolic dysfunction has been closely linked to anthracycline exposure following cardiotoxic treatment, and this systolic dysfunction can ultimately lead to heart failure [11]. Additionally, Mulrooney et al. found the prevalence of valvular and pericardial disease associated with radiation involving cardiac fields among childhood cancer survivors to be about 31% [12].

Many emerging studies are increasingly focusing on the various CV outcomes in childhood cancer survivors. According to Borkowski et al., social determinants of health are now recognized as key modulators of long-term outcomes. Survivors from lower socioeconomic backgrounds may experience delayed diagnosis of cardiac complications, limited access to specialized survivorship care, and higher cumulative risk due to coexisting lifestyle and environmental stressors [13]. Addressing these disparities is therefore important as survivorship continues to evolve. This review seeks to highlight some of these insights from available literature covering the years 2010 and 2024 on studies published, focusing on observational and retrospective studies on the long-term CV outcomes in childhood cancer survivors.

## Review

Materials and methods

Literature Search Strategy

The literature search was conducted in databases such as PubMed, ScienceDirect, and Web of Science following the guidelines underpinned by the International Prospective Register of Systematic Reviews (PROSPERO) and the Meta-analysis of Observational Studies in Epidemiology (MOOSE) group’s reporting guidelines. The literature search included keywords like “childhood cancer survivors”, “pediatric cancer survivors”, “adolescent cancer survivors”, “cardiovascular outcomes”, “cardiovascular health”, “heart disease”, “cardiac dysfunction”, “vascular complications”, “time frame”, “cancers treatments”, and “long-term cancer effects”. To discover publications on cancer epidemiology, the study employed a search approach that included a combination of keywords and subject headings derived from two earlier filters, as well as manual examination of popular articles and databases.

Eligibility Criteria

Two reviewers were involved in evaluating the eligibility of the included studies. In case there were differences between the two authors, a third independent author was involved, and the issue was resolved through discussion. The first step involved screening the study titles and abstracts. Research that was unique, involved human subjects, published in a peer-reviewed publication in the English language between the years 2010 and 2024, and pertinent to the study’s objectives was eligible for full-text review. The study included a general population showing charges after intervention, standardized outcomes, cross-sectional, prospective, observational studies, and patients who have survived for at least five years after diagnosis.

Exclusion involved studies with smaller sample sizes (less than 30), studies not addressing the outcome of interest, and studies not targeting the group of interest.

The qualifying criteria described above were designed to ensure that only up-to-date, relevant, and high-caliber research was used to provide meaningful insights into the long-term CV outcomes of children who have survived cancer. Disputes were quickly resolved through inclusive discussions that led to an agreement. The researchers retrieved and recorded all the data relevant to this review.

Data Extraction and Study Quality Assessment

The study used a preestablished data template to extract data from qualified research based on study characteristics, population, treatment regimen, follow-up period, and participant age. Mean values were used when median values were unavailable, and also the reported events. The quality of the studies was assessed using the Newcastle-Ottawa Scale (NOS) for nonrandomized studies (0-3: high risk of bias; 4-6: moderate risk; and 7-9: low risk), while randomized trials were evaluated using the Cochrane Risk of Bias Assessment Tool.

Statistical Analysis

The data was analyzed using the Review Manager (RevMan) 5.4.1 program. Due to the variation in the types of cancer and the treatments, it was impossible to carry out a meta-analysis, as the effect sizes of each study varied based on the type of cancer that was being treated and the treatments applied. Therefore, the statistical analysis of this systematic review entails evaluating the quality and risk of bias in the included research. The NOS instrument was developed to assess the risk of bias for nonrandomized studies. For randomized trials, the Cochrane risk of bias assessment tool was used to assess the risk of bias of items, with the main criteria being random sequence generation, allocation concealment, participant and personnel blinding, outcome assessment blinding, incomplete outcome data, selective reporting, and other biases. A funnel plot was used to assess the risk of publication bias, supported by Egger’s test statistics, with scores interpreted as follows: 0-2 indicating high risk of bias, 3-4 moderate risk, and 5-7 low risk.

Results

**Figure 1:**
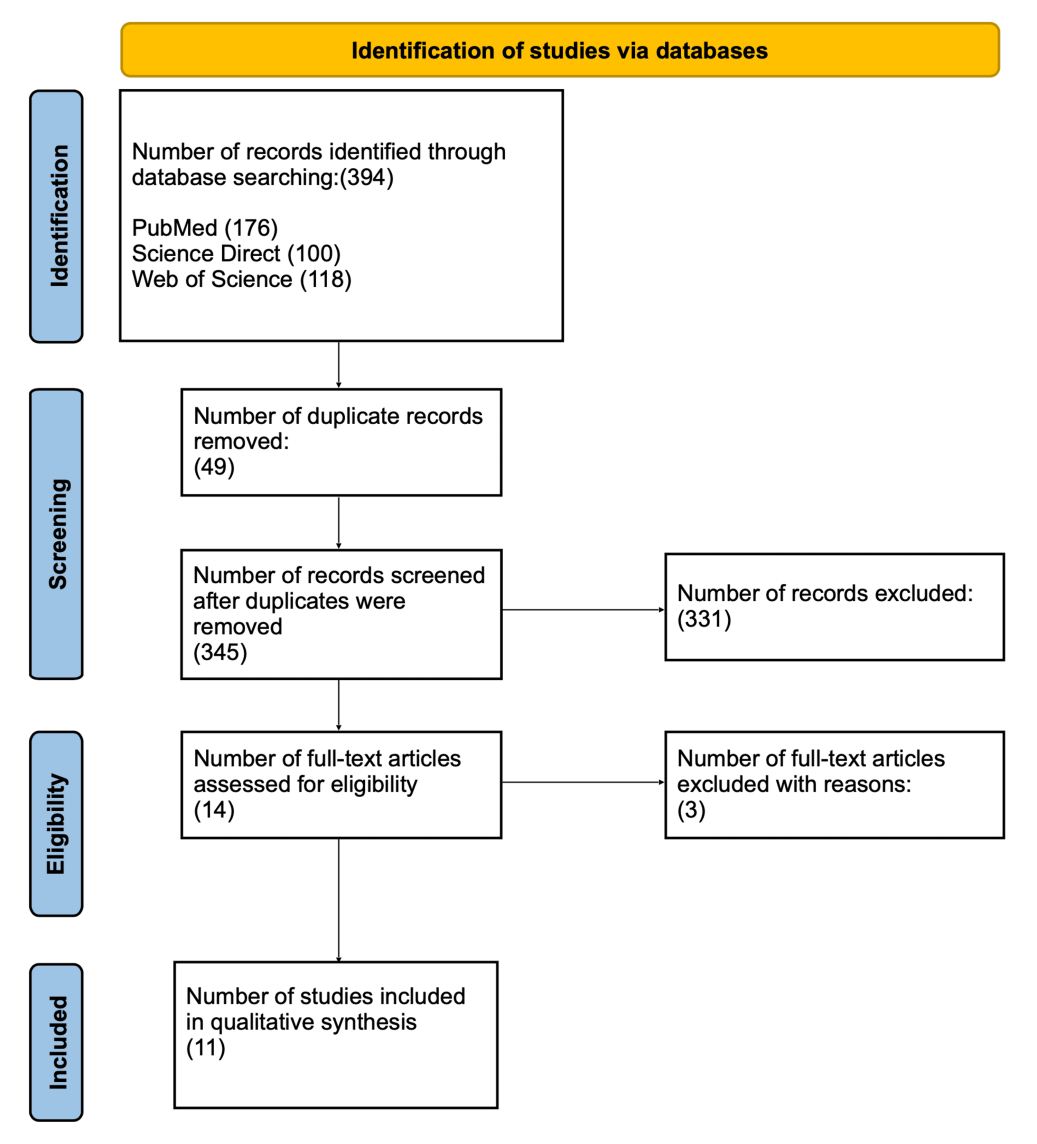
PRISMA flow diagram PRISMA, Preferred Reporting Items for Systematic reviews and Meta-Analyses

The initial search yielded 394 papers from multiple databases, including PubMed (176), ScienceDirect (100), and Web of Science (118). After 49 duplicate articles were excluded, 345 papers qualified for the screening process. During this stage, 331 articles were excluded. As a result, only 14 articles were considered for eligibility assessment. In this step, three more papers were excluded for reasons such as study design, lack of study sample, and lack of outcome of interest. Finally, only 11 papers were considered for the qualitative synthesis (Figure 1).

Table 1 lists the key characteristics of the papers that were selected for review. All 11 of the publications evaluated in this systematic review looked at the long-term CV outcomes of children who survived cancer. Every study that was part of the review was published in English between 2010 and 2024 and was carried out in different parts of the world.

**Table 1:** Study characteristics of articles included in systematic review (n = 11) ABPM, ambulatory blood pressure monitoring; AYA, adolescent and young adult; BCC, basal cell carcinoma; CCS, childhood cancer survivors; CV, cardiovascular; CVD, cardiovascular disease; CVRF, cardiovascular risk factor; GVHD, graft-versus-host disease; HCT, hematopoietic cell transplantation; HDL, high-density lipoprotein; MetS, metabolic syndrome; MM, multiple myeloma; NHB, non-Hispanic Black; NHW, non-Hispanic White; QoL, quality of life; RCT, randomized controlled trial

Author	Region	Study type	Type of cancer	Type of treatment	Cases with cancer	Control cases	Outcome/results	Conclusion
Mulrooney et al. (2016) [12]	USA	Cross-sectional study	Cardiomyopathy	Cardiotoxic therapy	1,853	None	Of the survivors, 7.4% had cardiomyopathy, 3.8% had coronary artery disease, 24.8% had valvular regurgitation or stenosis, and 4.4% had conduction or rhythm problems. Survivors aged 30-39 years had a 3-4% prevalence of cardiac problems, whereas those aged 40 and above had a 10-37% prevalence.	Significant subclinical illness was found in adult survivors of childhood cancer by CV screening.
Ha et al. (2024) [14]	Korea	Retrospective case-control study	Myeloma	Cardiotoxic chemotherapy, high doses of steroids, proteasome inhibitors, immunomodulatory drugs, and radiotherapy	15,402	15,402	The case cohort showed significantly lower CV events and risk at eight years, with higher risk among patients under 50 and a higher incidence of CVD at five years.	The study emphasizes the importance of regular CVD monitoring in patients with MM, especially those under 50 years old at the time of diagnosis.
Morel et al. (2020) [15]	Multiple facilities in Montreal, Canada	Cross-sectional analysis	Acute lymphoblastic leukemia	Cranial radiation therapy	146	246	Results showed high leptin-adiponectin ratios were associated with obesity, insulin resistance, and MetS. High C-reactive protein levels were linked to insulin resistance, dyslipidemia, and MetS.	The study reveals a direct link between lipopolysaccharide-binding protein and metabolic endotoxemia, inflammation, and cardiometabolic outcomes, suggesting potential biomarkers for prevention strategies.
Chow et al. (2022) [16]	United States	RCT	P9404 (acute lymphoblastic leukemia/lymphoma)	Dexrazoxane	1,066	242	Dexrazoxane did not correlate with CV mortality, all-cause mortality, recurrence, or second malignancies. There were no heart transplants or CV deaths among P9754 patients exposed to dexrazoxane. Cardiomyopathy rates did not change, although the incidence of serious CV events was lower with dexrazoxane (5.6%) than without it (17.6%).	Dexrazoxane did not appear to have any adverse effects on event-free survival, second cancer risk, or long-term mortality.
Chait-Rubinek et al. (2019) [17]	Australia	Retrospective study	Peripartum cardiac dysfunction	Anthracycline chemotherapy	64	65	Peripartum cardiac events were possible for long-term survivors who were less than 30 years old at the time of their cancer diagnosis and who had at least one pregnancy within five years of the diagnosis. A 55-fold higher incidence of peripartum cardiomyopathy was associated with 7.8% of the 64 eligible women experiencing peripartum cardiac episodes. Younger age and higher anthracycline dosage were risk factors.	In long-term survivors of pediatric and AYA cancers that have previously received cardiotoxic treatment, peripartum cardiac dysfunction is a rare but potentially dangerous side effect. Patients who are at risk should definitely have a peripartum cardiac evaluation.
Schoormans et al. (2018) [18]	Netherlands	Observational matched cohort study	Breast, prostate, non-Hodgkin, Hodgkin, lung and trachea, BCC, and colorectal cancer	Two drug dispenses of cardiac therapeutics (i.e., ATC code C01) at unique dates within six months or hospitalization for CVD (ICD-9 codes 410-414 and 420-429)	Breast = 6,762, prostate = 4,504, non-Hodgkin = 1,553, Hodgkin = 173, lung and trachea = 2,661, BCC = 12,476, and colorectal cancer = 4,628	Breast = 6,762, prostate = 4,504, non-Hodgkin = 1,553, Hodgkin = 173, lung and trachea = 2,661, BCC = 12,476, and colorectal cancer = 4,628	Even after taking into account conventional risk variables and treatment information, survivors of prostate and lung cancer still have a considerably increased risk of getting CVD when compared to cancer-free controls.	Compared to cancer-free controls, cancer survivors are more likely to experience incident CVD, necessitating lengthier follow-up studies to assess long-term risk.
Noyd et al. (2023) [19]	USA	Retrospective cohort study	Leukemia, CNS malignancy, Hodgkin lymphoma, non-Hodgkin lymphoma, Wilms tumor, neuroblastoma, soft tissue sarcoma, or a bone tumor	Chemotherapy agents, cyclophosphamide, and anthracycline doses	NHB = 1,092, Hispanic = 1,405	NHW = 13,960	When compared to NHW survivors, NHB and Hispanic survivors had greater cumulative rates of diabetes, obesity, multiple CVRFs, and hypertension by the age of 40. Hispanic survivors were more likely to have diabetes and obesity, and these survivors were more likely to have hypertension, obesity, and numerous CVRFs.	Comparing NHB and Hispanic survivors to NHW survivors, the greater burden of CVRFs was comparable to that of the general population. In this population at high risk, it is imperative to promote CV health equity.
Friedman et al. (2017) [20]	New York	Retrospective analysis	Leukemia or lymphoma	Total body irradiation	123	145	According to the study, a higher cumulative incidence of CVRF clusters over time was linked to raised blood pressure, hyperglycemia, low HDL, hypertriglyceridemia, and obesity. The likelihood of developing a CVRF cluster was linked to factors such as growth hormone deficit, cranial radiation history, and grade II-IV acute GVHD.	The necessity of frequent screening in children is highlighted by the fact that HCT survivors had low HDL prevalence and increased hypertriglyceridemia but no glucose intolerance, elevated blood pressure, or CVRF cluster.
Ernst et al. (2023) [21]	Germany	Retrospective cohort study	Leukemia, lymphomas, and CNS tumors	Antineoplastic treatment	663	975	Higher symptom burden and worse functional QoL are experienced by CCS, especially women. Health risk factors are linked to lower QoL, while younger age, education, marriage, and participation in active sports are linked to improved QoL.	Long-term CCS reported lower QoL than the comparator sample in every domain. Long-term monitoring and health promotion are critically needed, as evidenced by the unfavorable correlations with risk variables and physical ailments.
Hsiao et al. (2023) [22]	USA	Retrospective chart review	Leukemia/myelodysplastic syndrome, neuroblastoma, and Wilms tumor	Longitudinal ABPM, echocardiography, serum creatinine, and first-morning urine protein/creatinine ratios	130	422	According to the study, the most frequent diagnoses were Wilms tumor, neuroblastoma, and leukemia/myelodysplastic syndrome. The majority of patients had proteinuria, hypertension, and poor kidney function. Several ABPMs lowered blood pressure loads; patients with hypertension received stem cell transplantation or total body irradiation, while those with compromised kidney function received ifosfamide.	Nephrology frequently saw survivors with a history of neuroblastoma, Wilms tumor, and leukemia/myelodysplastic syndrome. When hypertension was more widely recognized and treated, CV measures significantly improved.
Winther et al. (2018) [23]	Denmark, Finland, Iceland, Norway, and Sweden	Retrospective cohort study	CNS tumor, leukemia, lymphoma, and solid tumor	Four time periods (zero to four, five to nine, 10-14, and 15-19 years) and diabetes	33,160	212,892	Of the 29,324 one-year survivors, 324 had been diagnosed with diabetes, and 2108 had been diagnosed with CVD. Compared to comparison patients, survivors were 1.7 times more likely to get diabetes, and those who already had diabetes were 2.4 times more likely to have CVD.	Compared to CCS without diabetes, those with diabetes have a much higher risk of CVD. Diabetes, however, does not raise survivors’ risk of CVD above that of the general population.

Assessment of Item Risk of Bias of the Retrospective Studies

The quality of the retrospective, cross-sectional, and observational studies was assessed using the NOS. Each study could receive up to one star for each item in the selection and outcome categories, while the comparability category allowed for a maximum of two stars. Based on the assessment, eight out of the 10 included studies were rated as high quality with minimal risk of bias [12,14,15,18-21,23]. Two studies were found to have a moderate risk of bias [17,22], and none were classified as having a high risk of bias. Overall, the included studies were of good quality (Table 2), supporting the reliability of the findings presented in this review.

**Table 2:** Assessment of quality of the included studies Each study received one star (*) for every numbered item in the selection and outcome categories. The comparability category, however, could receive up to two stars (**). According to the evaluation results, “*” indicates a low risk of bias in the selection and outcome domains, “**” reflects a low risk of bias in comparability, while “-” denotes a high risk of bias.

Author	Selection				Comparability	Outcome			Total score (0-3: high risk, 4-6: moderate risk, and 7-9: low risk)
	Representativeness of the exposed cohort	Selection of the nonexposed cohort	Ascertainment of exposure	Demonstration that the outcome of interest was not present at the start of the study	Comparability of cohorts on the basis of the design or analysis	Assessment of outcome	Was the follow-up long enough for outcomes to occur	Adequacy of follow-up of cohorts	
Mulrooney et al. (2016) [12]	*	*	*	*	**	*	*	*	9/9
Ha et al. (2024) [14]	*	*	*	*	**	*	*	*	9/9
Morel et al. (2020) [15]	*	*	*	*	**	*	*	*	9/9
Chait-Rubinek et al. (2019) [17]	*	*	*	-	*	*	-	-	5/9
Schoormans et al. (2018) [18]	*	*	*	*	**	*	*	*	9/9
Noyd et al. (2023) [19]	*	*	*	-	**	*	*	-	7/9
Friedman et al. (2017) [20]	*	*	*	*	**	*	*	*	9/9
Ernst et al. (2023) [21]	*	*	*	*	**	*	*	*	9/9
Hsiao et al. (2023) [22]	*	*	*	-	*	*	-	-	5/9
Winther et al. (2018) [23]	*	*	*	-	**	*	*	-	7/9

Assessment of Item Risk of Bias of the Randomized Controlled Trial (RCT) Studies

**Figure 2:**
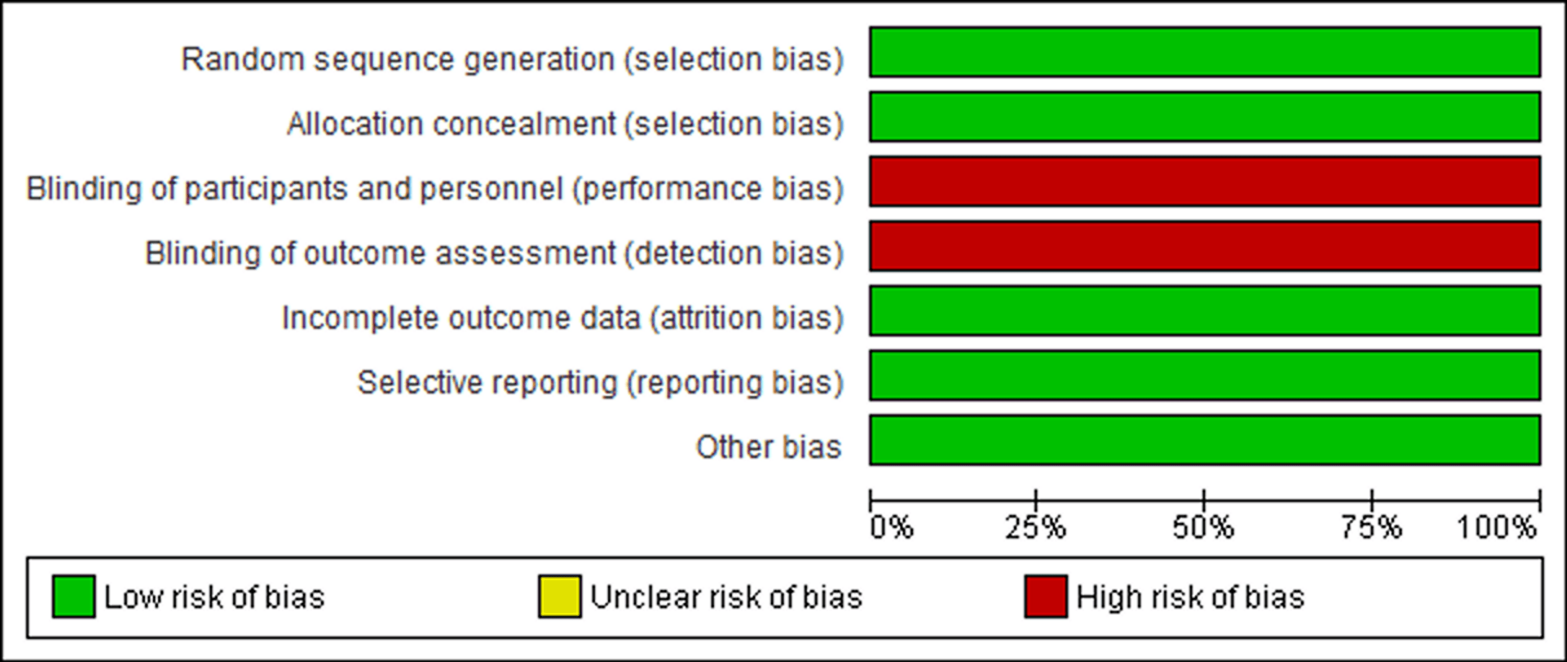
Summary of studies’ risk of bias of each item Scores were interpreted as follows: 0-2 indicated a high risk of bias, 3-4 a moderate risk, and 5-7 a low risk of bias.

**Figure 3:**
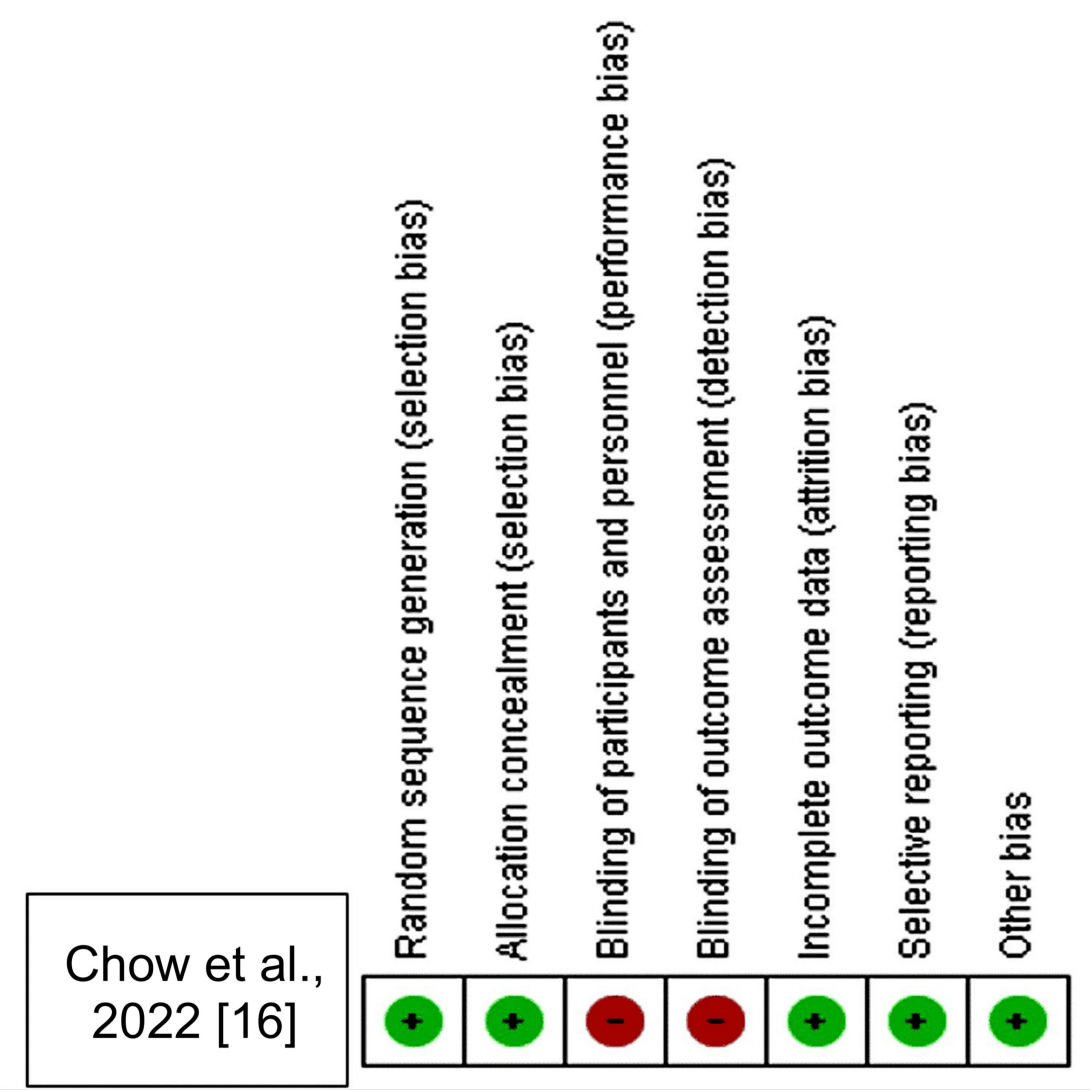
Summary of each study’s risk of bias for each item Based on the reviewers’ evaluation of the study items’ risk of bias, the risk-of-bias summary displays red circles for high risk, green circles for low risk, and white circles for uncertain risk.

The RCT considered in this analysis was of high quality and had low bias risk (Figure 2, Figure 3). Out of seven items assessed for possible risk of bias, only 2/7 items had a high risk of bias, while 5/7 had a low risk of bias. Generally, the RCT included in this systematic review was of good quality, implying that the findings of this study can be reported with confidence.

Assessment of Publication Bias of the Included Studies

**Figure 4:**
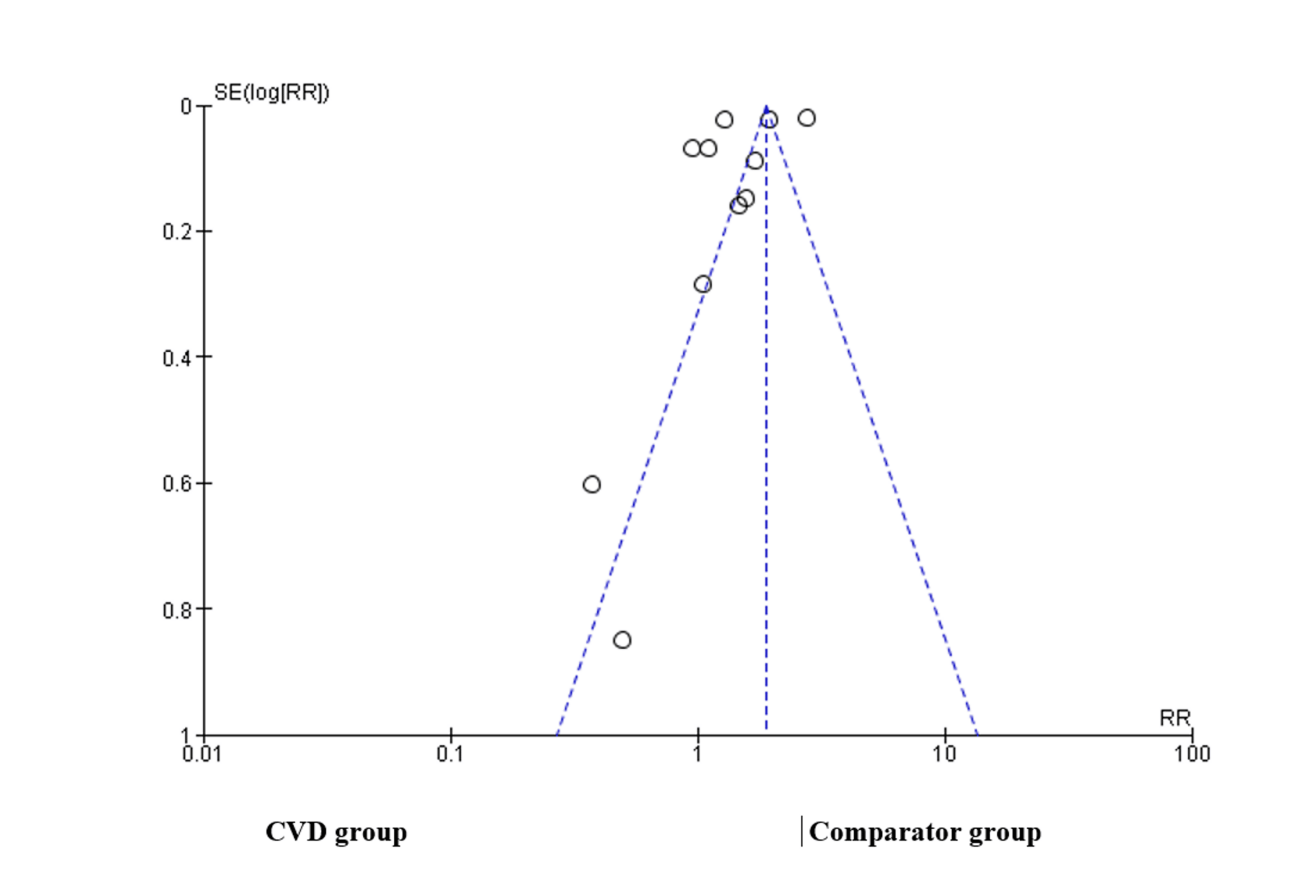
Funnel plot depicting publication bias x-axis: standard error; y-axis: effect size CVD, cardiovascular disease; RR, relative risk; SE, standard error

Egger’s test was used to assess the risk of publication bias. The results show that more studies and outliers are on the left side of the funnel plot, which displays an asymmetric distribution of effect sizes as a function of study precision. This suggests that the number of studies on either side is disproportionate. This implies that there may be a possibility of publication bias in the CVD group (Figure 4).

Discussion

In evaluating the long-term CV outcomes of pediatric cancer survivors, this systematic review analysis identified a number of risk factors for CVD and possible management strategies. According to a study by Ha et al., when compared to the control group, the case cohort’s incidence of CV events and overall risk for CVD was significantly reduced over an eight-year period (12.5% vs. 22.1%). Additionally, a five-year follow-up verified that the case group had a decreased incidence of CVD (7.8% vs. 9.8%). However, despite the general benefits seen in the community, patients under 50 years old in the case cohort had a noticeably increased risk of CVD, suggesting that age-specific factors may affect outcomes [14].

In a study by Morel et al., it was revealed that there is a significant correlation between cardiometabolic risk factors and markers of inflammation, oxidative stress, and endothelial dysfunction in children who have survived childhood acute lymphoblastic leukemia (cALL) (p < 0.05). In particular, obesity, insulin resistance, and metabolic syndrome were all strongly predicted by a high leptin-adiponectin ratio. Obesity was also linked to elevated levels of TNF-α and PAI-1, whereas insulin resistance, dyslipidemia, and metabolic syndrome were linked to elevated levels of CRP. Further, the study noted that inflammation plays a key role in the long-term health concerns for cALL survivors by mediating the relationship between endotoxemia and cardiometabolic problems [15]. Further, Chow et al. reported that dexrazoxane does not raise the incidence of relapse, second malignancies, or mortality during long-term follow-up when administered in conjunction with doxorubicin in the treatment of pediatric cancer. Crucially, although rates of cardiomyopathy alone were not significantly different, individuals who received dexrazoxane experienced adverse CV events that were less prevalent (5.6%) than those who did not (17.6%). Additionally, the study noted that the cohort receiving P9754, a high dose of dexrazoxane, did not experience any heart transplants or CV fatalities. Hence, dexrazoxane was found to be safe for CVD over the long term and may help prevent more widespread CV problems [16].

According to research by Chait-Rubinek et al., female cancer survivors have a significantly elevated risk of peripartum cardiomyopathy, with an incidence that is 55 times higher than that of the general population. The majority of the included participants did not regain their prepartum cardiac function, and 7.8% of them had peripartum cardiac episodes. Higher anthracycline dosages and younger age at diagnosis seemed to increase risk. In contrast to noncancer controls, survivors of prostate and lung/trachea cancers had considerably higher CVD risk, with lung/trachea cancer survivors continuing to have this risk even after controlling for treatment history and standard CVD variables [17]. Schoormans et al. found that compared to the participants without cancer, survivors of lung/trachea and prostate malignancies are more likely to acquire CVD. Survivors of lung and trachea cancer had a significantly higher risk of CVD even after controlling for conventional risk variables and treatment specifics. For survivors of prostate cancer, the higher risk of CVD was most noticeable for those receiving hormone therapy and those who did not already have CV risk factors (CVRFs) [18]. In addition, a study by Noyd et al. found that among childhood cancer survivors by the age of 40, there are notable racial and ethnic differences in cardiometabolic health. Compared to non-Hispanic White survivors, non-Hispanic Black (NHB) and Hispanic survivors had greater cumulative incidences of diabetes, obesity, hypertension, and numerous CVRFs. While Hispanic survivors had higher rates of diabetes and obesity, NHB survivors had significantly higher risks of hypertension, obesity, and multiple CVRFs after controlling for sociodemographic and therapeutic factors [19].

Moreover, a study by Friedman et al. reported that childhood survivors of total body irradiation have a considerable long-term risk of acquiring cardiometabolic problems. Within 10 years, over two-thirds developed hypertriglyceridemia, and more than half had low HDL. Over time, there was a noticeable increase in the cumulative incidence of CVRF clusters. Furthermore, growth hormone deficit, previous cranial radiation, and moderate to severe acute graft-versus-host disease were found to be significant predictors of the development of clustered CVRFs. The study emphasizes the importance of early management and proactive CV surveillance in this high-risk group [20]. In a study by Ernst et al., it was revealed that compared to the general population, childhood cancer survivors, especially girls, have a considerably lower functional quality of life (QoL) and a higher burden of symptoms. The study also reported that better overall QoL was associated with younger age, greater education, marriage, and regular physical exercise in the childhood cancer survivor group (p < 0.05). On the other hand, physical diseases like CVD and health risk factors, including dyslipidemia and physical inactivity, were linked to lower QoL. The study highlighted the need for focused assistance and health promotion strategies to enhance the long-term health of childhood cancer survivor patients, particularly women [21]. On the other hand, Hsiao et al. found that at a median of eight years following treatment, 68% had reduced kidney function, 12% had proteinuria, and 38% had hypertension. Further, blood pressure monitoring significantly improved for those who received repeated ABPMs. Notably, survivors who had received total body irradiation or allogeneic stem cell transplantation were more likely to have hypertension, while prior ifosfamide treatment was significantly associated with reduced kidney function [22].

According to Winther et al., childhood cancer survivors had significantly higher long-term health risks. The study revealed that within 15 years of diagnosis, survivors were 3.6 times more likely to develop CVD and 70% more likely to develop diabetes than noncancer patients in the control group. The study also found that even after reaching the age of 15, their risk of CVD was nearly doubled. It also found that diabetes significantly raised the risk of CVD in both survivors and the general population by 2.4 times. The finding of this study shows that there is a need for childhood cancer survivors to receive preventative care and ongoing cardiometabolic monitoring well into adulthood [23]. The study by Mulrooney et al. found that among long-term childhood cancer survivors, the majority of whom were asymptomatic, bear a heavy burden of undetected heart defects. The most prevalent conditions included cardiomyopathy (7.4%), coronary artery disease (3.8%), conduction problems (4.4%), and valve disease (28%). Numerous examples were discovered during the assessment. Heart diseases became more common as people aged, particularly beyond the age of 40. The risk of cardiomyopathy was considerably raised by exposure to high-dose anthracyclines (≥250 mg/m²) and cardiac radiation, but the risk of valvular abnormalities was highest when radiation exposure was >1,500 cGy and anthracyclines were combined. On that basis, the study highlighted the necessity of cardiac monitoring throughout life [12].

Andreescu found that serum biomarkers may be useful for CV risk assessment in patients with hematological malignancies. The study revealed that in order to prevent long-term CV effects, these biomarkers may be able to identify early signs of heart damage, direct therapeutic choices, and customize treatment plans [24]. The study by Albulushi et al. revealed that the risk of cardiometabolic diseases, such as diabetes, dyslipidemia, and hypertension, is markedly increased among cancer survivors. Up to 60% of survivors suffer from hypertension, which is frequently brought on by vascular damage from therapy, psychological stress, and preexisting illnesses. Moreover, the study found that the common causes of dyslipidemia include poor diet, decreased physical exercise, and cancer treatments. In addition, metabolic syndrome, obesity, and chronic inflammation increase the risk of CVD. Therefore, the study suggested that in order to improve long-term health outcomes for cancer survivors, there is a need for addressing these problems by having dietary modifications, consistent exercise, and emotional support [25]. Zhao et al. found that one significant late effect of hematopoietic stem cell transplantation is CVD, which is more common in long-term survivors. The major risks associated with this disease include cardiotoxic therapies, conditioning programs, and post-transplant problems. On that basis, this study noted that determining treatment options and supportive care techniques requires assessing heart function both before and after transplantation [26]. This implies that there are a number of CV risks among childhood cancer survivors based on the type of cancer, treatment method, period, and other demographic factors. Therefore, there is a need for an approved strategy for managing these risks among the affected population in order to mitigate the effects of the disease.

Limitations

This systematic review found a number of limitations. First of all, it made extensive use of observational and retrospective research, which might be biased by selection and recollection. Second, there may have been discrepancies in the combined results due to variations in study designs, cancer types, treatment regimens, and follow-up times. Furthermore, although the majority of the studies were of excellent quality, some had moderate risks of bias, which could have an impact on the overall findings. The publication bias revealed by the study further restricts the generalizability of the study’s findings. Finally, the demographic and geographic differences among the included studies limit the application of the findings to all groups.

## Conclusions

Long-term risks of CV and cardiometabolic diseases are significantly higher for childhood cancer survivors. These risks are influenced by the kind of cancer, the type of therapy (e.g., anthracyclines and radiation), and demographic factors such as age, gender, and ethnicity. Despite having significant underlying cardiac issues, many survivors are asymptomatic, highlighting the critical significance of lifelong CV surveillance. Therefore, there is a need to put in place systematic, lifelong programs for cardiometabolic screening and risk reduction that are tailored to each person’s risk profile. These programs should include echocardiography, regular serum biomarker testing, lifestyle modifications (exercise and nutrition), and psychosocial support. On that basis, this study suggests future studies focus on creating age- and treatment-specific cardioprotection regimens, examining the long-term impacts of preventive medications like dexrazoxane, and evaluating the contribution of personalized medicine, which includes genetic and biomarker profiling, to the management and prediction of CV risk.
